# Rare nonunion of tibial plateau fracture with patellar tendon rupture: A case report

**DOI:** 10.1097/MD.0000000000045622

**Published:** 2025-11-07

**Authors:** Tao Xiong, Zhou Feng Fan, Wu Zeng

**Affiliations:** a ShangRao Guangxin District People’s Hospital, Jiangxi, China; b Suichang County People’s Hospital, Suichang, Zhejiang Province, China; c Suichang County People’s Hospital, Suichang, Zhejiang Province, China.

**Keywords:** case report, nonunion, patellar tendon rupture, tibial plateau fracture

## Abstract

**Introduction:**

Tibial plateau fractures (TPFs) are often complex, resulting from high-energy mechanisms of injury, such as being struck by a heavy object, falling from a height, or colliding with a motor vehicle. Due to the increasing geriatric population, TPFs have become more common, comprising approximately 8% of all fractures in patients aged > 65 years, and complex patterns are defined as Schatzker V and VI. Patellar tendon avulsion from the tibial tubercle is one of the most common and serious forms of extensor mechanism rupture; however, TPFs associated with rupture of the patellar tendon are extremely rare, with only one case being reported in the literature. TPF nonunion is relatively rare and has various causes, including age, injury severity, fracture classification, and the presence or absence of important diseases, such as anemia and hypoproteinemia. As observed, no case associated with patellar tendon rupture, and TPF nonunion has been reported previously. Herein, we report the first such case.

**Patient concerns:**

A 74-year-old man with no history of drug allergies, chronic diseases, or family diseases was injured in a car accident on August 3, 2021. The patient was referred to the emergency department for anterior knee pain, bleeding, chest pain, and abdominal pain that prevented him from standing and walking.

**Therapeutic process:**

The wound surface in the anterior knee was approximately 5 cm × 6 cm, which exhibited soft-tissue defects with bone exposure. Radiographs were obtained and showed a tibial plateau comminuted fracture, classified as Schatzker type V. Subsequently, postoperative radiography showed that the plateau articular surface had achieved good reduction, and the metallic anchor and hollow tension screws were rigidly fixed. Fracture reduction was observed, and the implant position was quite similar to previous radiographs. Computed tomography (CT) showed a small bony callus in the fracture area approximately 3 months after surgery. CT revealed delayed fracture healing, with some patients also having deformities approximately 12 months after surgery.

**Outcomes:**

When reviewed after 2 years, the patient had no significantly pain and did not use walking aids. Knee function was preserved, with full extension and flexion to 90°. CT revealed TPF nonunion.

## 1. Introduction

Tibial plateau fractures (TPFs) are often complex, resulting from high-energy mechanisms of injury, such as being struck by a heavy object, falling from a height, or motor vehicle collisions. With the increase of in the geriatric population, TPFs have become more common, comprising approximately 8% of all fractures in patients older than 65 years,^[1,2]^ and complex patterns are defined as Schatzker V and VI.^[3,4]^ Patellar tendon avulsion from the tibial tubercle is one of the most common and serious forms of extensor mechanism rupture^[5]^; however, TPFs associated with rupture of the patellar tendon are extremely rare as only one case has been reported in the literature.^[6]^

Nonunion of TPFs is relatively rare and may be caused due to age,^[7]^ injury severity,^[8]^ fracture classification, and the presence or absence of important diseases, such as anemia and hypoproteinemia. As observed, no case associated with patellar tendon rupture, and TPF nonunion has been reported previously in medical literature. The following might be the first such case.

## 2. Case presentation

**Figure 1.**
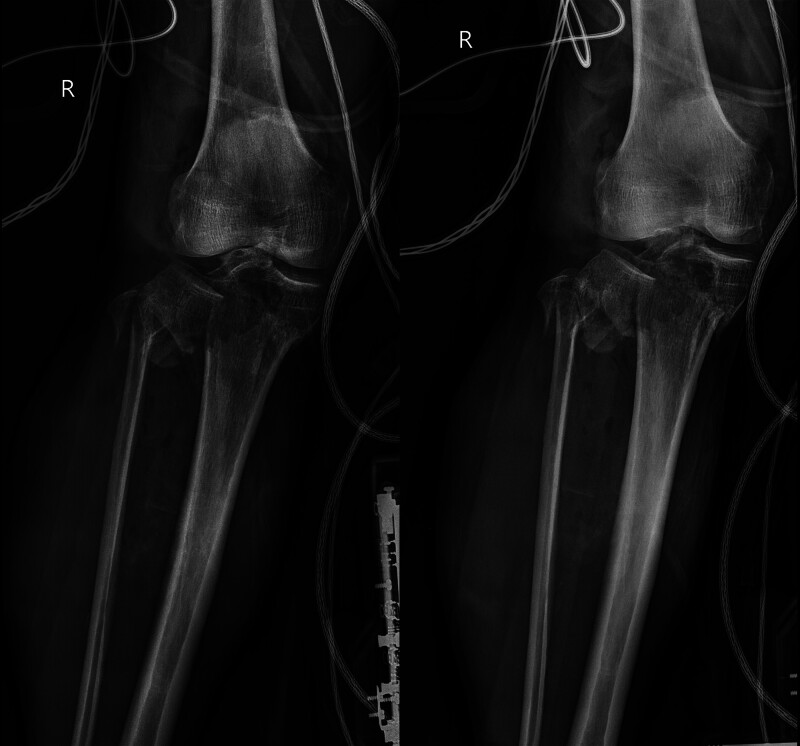
Radiographs showed a tibial plateau comminuted fracture of Schatzker type V (Fig. 1; date of examination: August 3, 2021). (A) Plain x-ray of the knee with anteroposterior view. (B) Plain x-ray of the knee with lateral view.

**Figure 2.**
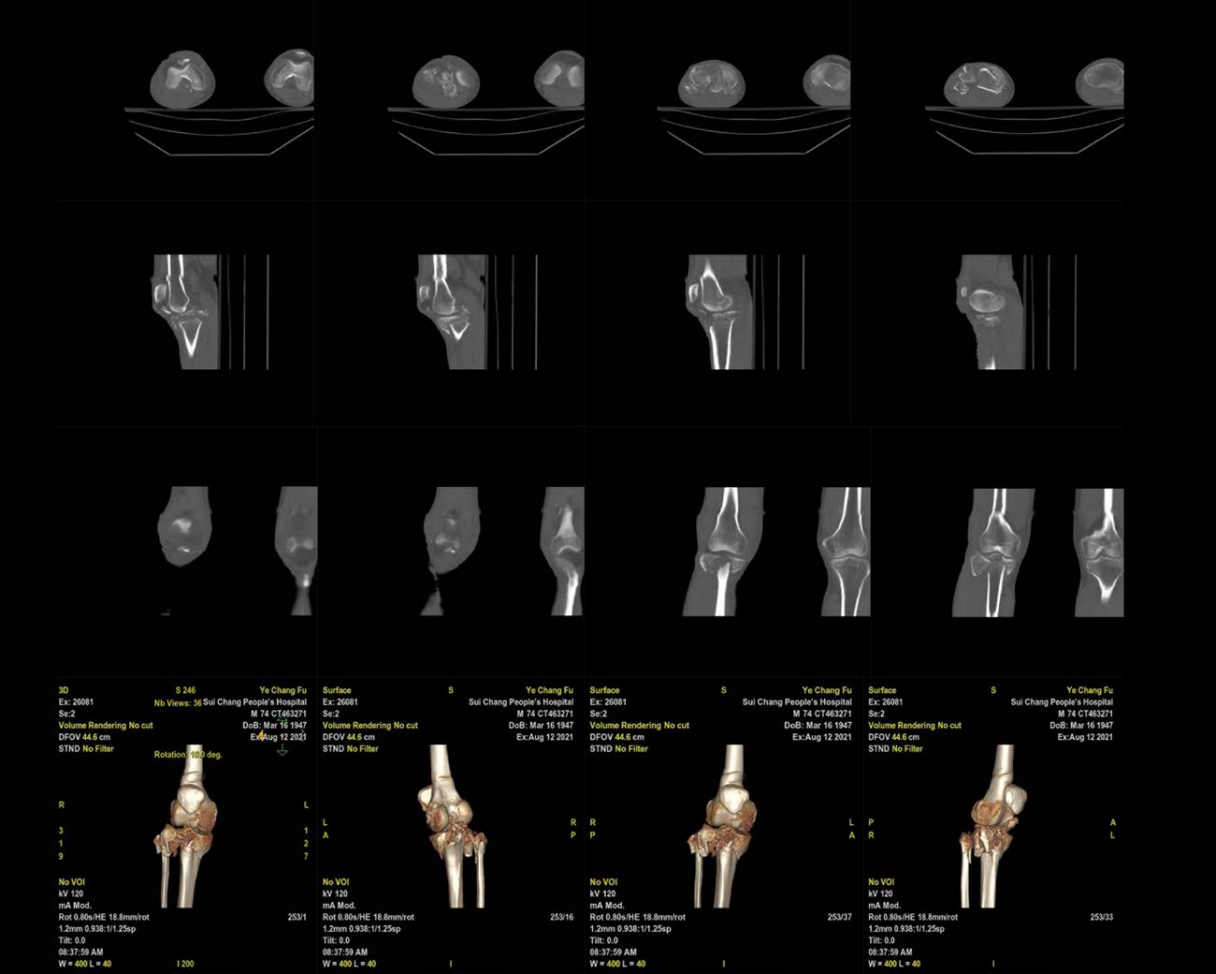
Computed tomography (CT) (Fig. 2; date of examination: August 12, 2021) revealed a clear traumatic hepatic rupture. Axial (A; B; C; D), agittal (E; F; G; H), coronal (I; J; K; L), and 3-dimensional (M; N; O; P) CT.

A 74-year-old man with no history of drug allergies, chronic diseases, or relevant family history sustained injuries in a car accident on August 3, 2021. The patient was referred to the emergency department for anterior knee pain, bleeding, chest pain, and abdominal pain that prevented him from standing and walking. The wound surface of the anterior knee was approximately 5 cm × 6 cm, which exhibited a soft tissue defect with bone exposure (The study has received ethical approval. It is supervised by the Ethics Committee of Suichang County People’s Hospital.). Radiographs showed a tibial plateau comminuted fracture of Schatzker type V (Fig. 1; date of examination: August 3, 2021). Computed tomography (CT) (Fig. 2; date of examination: August 12, 2021) revealed a clear traumatic hepatic rupture. Partial hepatic resection for the traumatic hepatic rupture was performed, and the open wounds of the knee were debrided and sutured in the emergency operating room. The skin, soft tissues, and whole body condition significantly improved after 10 days. The bone fractures were treated using open reduction and internal fixation through the traditional anterolateral and anteromedial regions of the knee joint. In addition, patellar ligament rupture with an avulsion fracture of the tibial tubercle was observed during surgery. The patellar tendon was repaired using a metallic anchor and 2 hollow tension screws. As a result of multiple transfusions and human albumin infusions after the operation, the patient’s acute blood loss anemia and serious hypoproteinemia has been markedly improved within 1 week.

**Figure 3.**
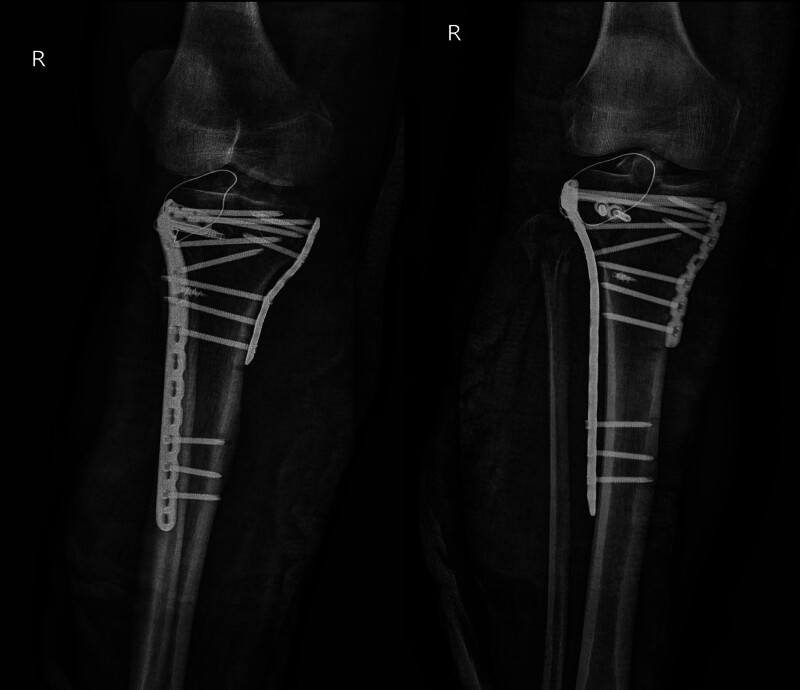
Postoperative radiography showed that the plateau articular surface achieved good reduction, and the metallic anchor and hollow tension screws were rigidly fixed (Fig. 3; date of examination: August 15, 2021). (A) Plain x-ray of the knee with anteroposterior view. (B) Plain x-ray of the knee with lateral view.

**Figure 4.**
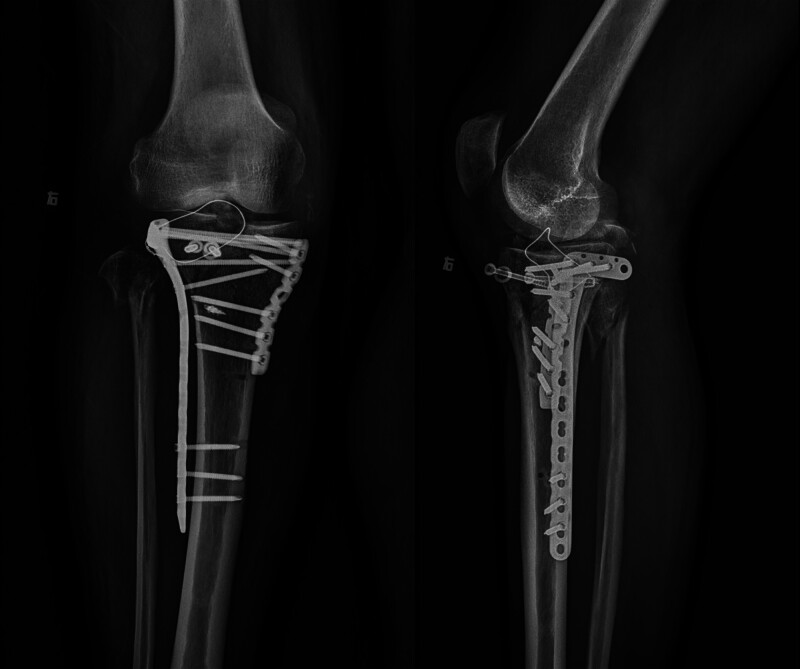
Postoperative radiographs showed fracture reduction was observed, and the implant position was similar to previous radiographs (Fig. 4; date of examination: September 25, 2021). (A) Plain x-ray of the knee with anteroposterior view. (B) Plain x-ray of the knee with lateral view.

**Figure 5.**
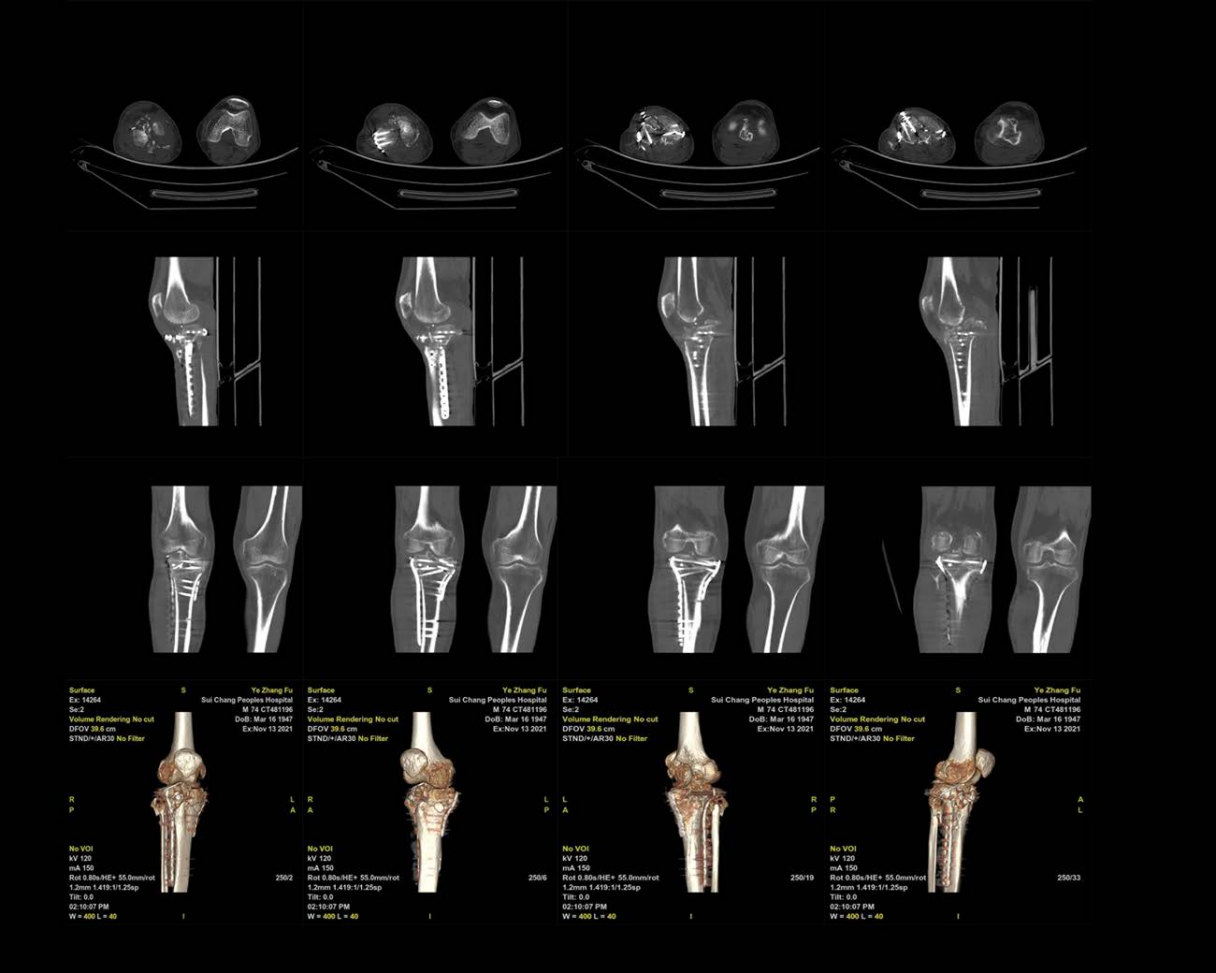
Computed tomography showed a small bony callus in the fracture area approximately 3 months after surgery (Fig. 5; date of examination: November 13, 2021). Axial (A; B; C; D), agittal (E; F; G; H), coronal (I; J; K; L), and 3-dimensional (M; N; O; P) CT.

Subsequently, postoperative radiography showed that the plateau articular surface achieved good reduction, and the metallic anchor and hollow tension screws were rigidly fixed (Fig. 3; date of examination: August 15, 2021). Additionally, fracture reduction was observed, and the implant position was similar to the previous radiographs (Fig. 4; date of examination: September 25, 2021). CT showed a small bony callus in the fracture area approximately 3 months after surgery (Fig. 5; Date of examination: November 13, 2021).

**Figure 6.**
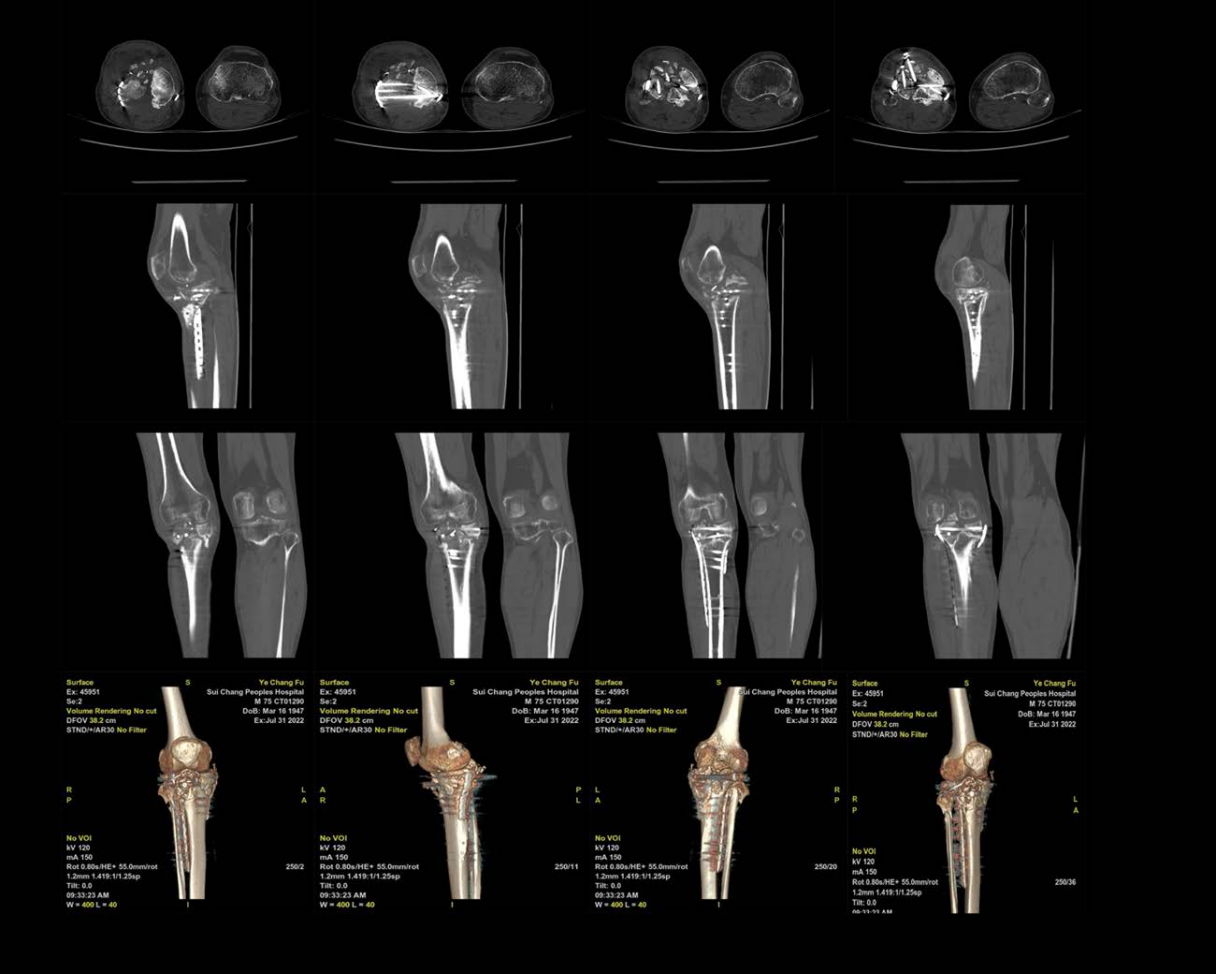
Computed tomography revealed delayed fracture healing, with some deformities approximately 12 months after surgery (Fig. 6; date of examination: July 31, 2022). Axial (A; B; C; D), agittal (E; F; G; H), coronal (I; J; K; L), and 3-dimensional (M; N; O; P) CT.

**Figure 7.**
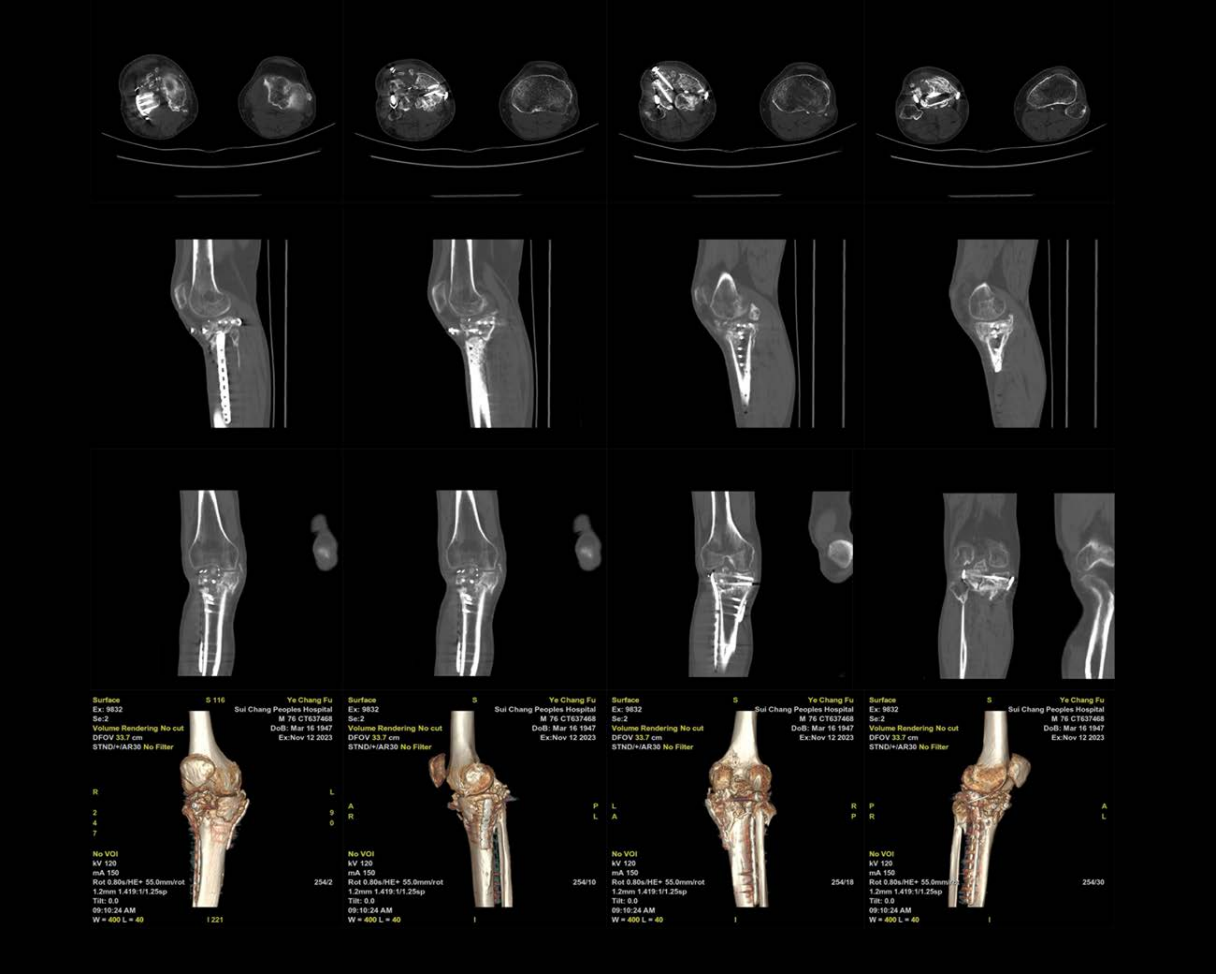
Computerized tomography revealed revealed nonunion of the TPF in this patient (Fig. 7; date of examination: November 12, 2023). Axial (A; B; C; D), agittal (E; F; G; H), coronal (I; J; K; L), and 3-dimensional (M; N; O; P) CT.

**Figure 8.**
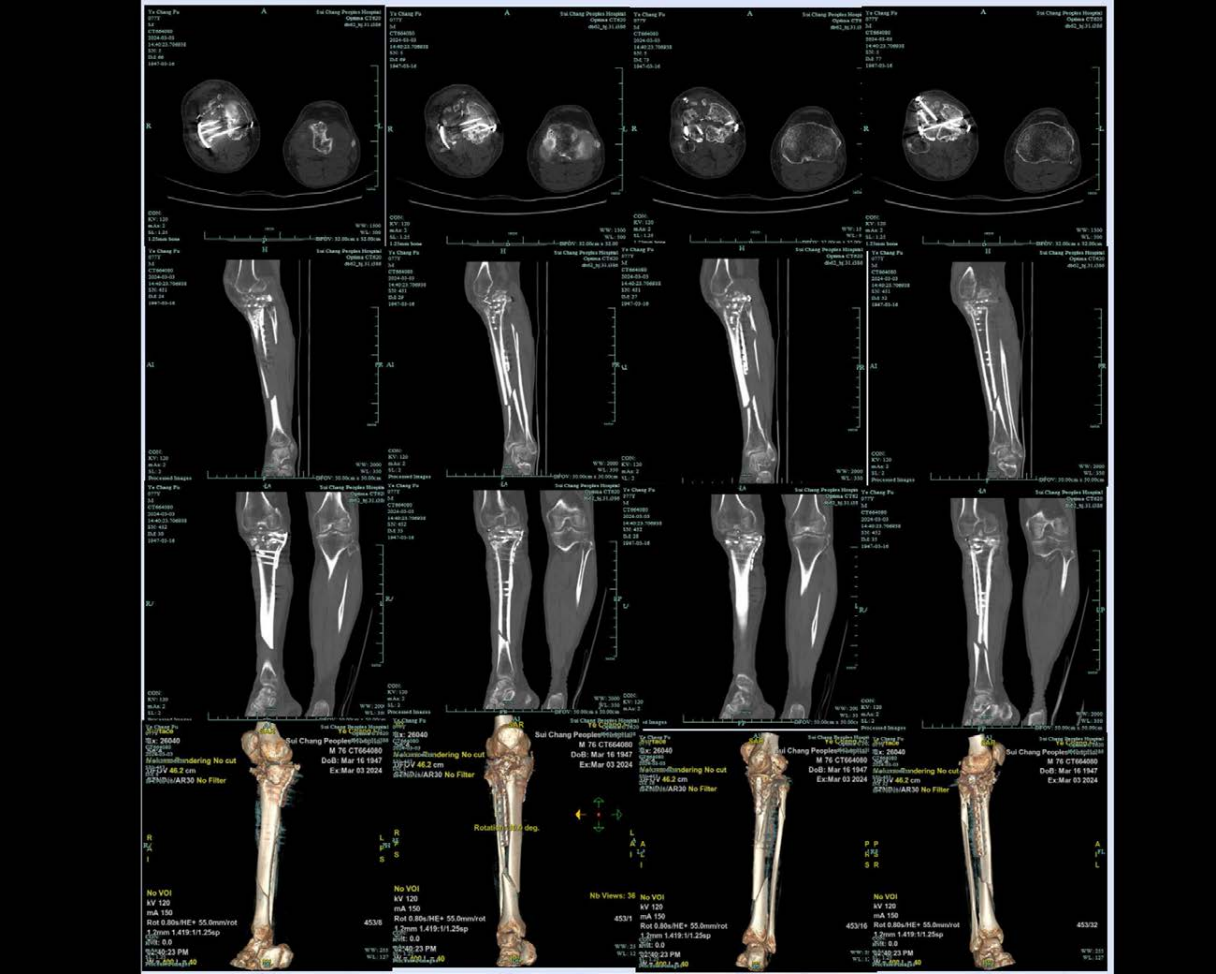
Computerized tomography showed fracture of the tibia and fibula with persistent nonunion of the TPF (Fig. 8; date of examination: March 3, 2024). Axial (A; B; C; D), agittal (E; F; G; H), coronal (I; J; K; L), and 3-dimensional (M; N; O; P) CT.

**Figure 9.**
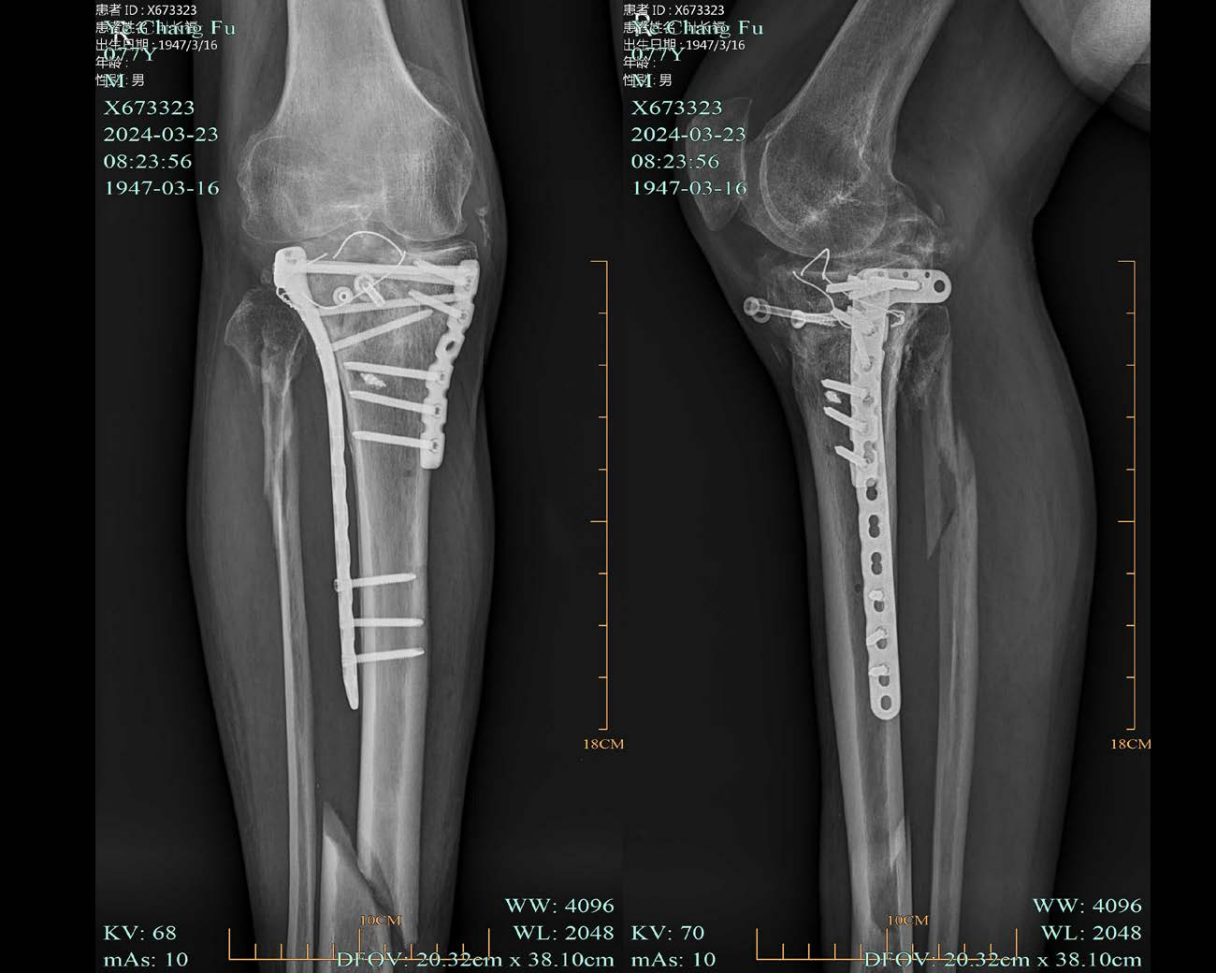
Radiography scan showed wire snap and loosened screw (Fig. 9; date of examination: March 23, 2024). (A) Plain x-ray of the knee with anteroposterior view. (B) Plain x-ray of the knee with lateral view.

Postoperatively, the right knee joint was fixed in an extended position with plaster for 2 weeks (Visual Analogue Score, 3 points/Mild pain), and an adjustable knee brace was used at 10°per week from 30°flexion to 90°over 6 weeks (Visual Analogue Score, 2 points/Mild pain). Three months after surgery, the patient initiated partial weight bearing and gradually advanced to full weight bearing by 6 months (Knee Society Score, 63 points). CT revealed delayed fracture healing, with some deformities approximately 12 months after surgery (Fig. 6; date of examination: July 31, 2022). After 2 years, the patient had no significantly pain and did not use walking aids on level ground, and the patient could fully extend and flex the knee at a 90°angle (Knee Society Score, 66 points). However, the patient experienced significant pain when going upstairs or downstairs. What’s more, CT revealed nonunion of TPF in this patient (Fig. 7; date of examination: November 12, 2023). Subsequently, the patient was referred to the emergency department for the right leg pain after a fall, which prevented him from standing and walking. CT showed fracture of the tibia and fibula with persistent nonunion of the TPF (Fig. 8; date of examination: 2024.03.03). Radiography scan (Fig. 9; date of examination: March 23, 2023) showed wire snap and loosened screw.

## 3. Discussion

Most clinical studies on the nonunion of TPFs have been derived from case reports or retrospective reviews.^[8–14]^ Though high-energy trauma was considered one of the most important factors in the nonunion of tibial plateau, a review article found that low-energy Schatzker class I and II constituted respectively 3.2% and 9.7% of nonunions. Therefore, some low-energy plateau fractures may be at risk for nonunion, regardless of the primary treatment.^[11,15–17]^

Severe or complex TPFs are difficult to treat and are often accompanied by various complications.^[8–10]^ Although the complication rates for high-energy TPFs treated surgically have been reported up to 30%, tibial plateau nonunions represent only 1% to 2% of TPF complications. Moreover, 52.2% of nonunions were associated with high-energy trauma and 54.8% of patients were Schatzker class VI.^[11,17]^

Fracture healing is a very complex process that involves blood supply, bony contact, adequate mechanical stability, and appropriate endocrine and metabolic processes.^[18]^ The severity of the bone comminution and soft-tissue lesion is positively associated with high-energy trauma.^[15,16]^ Additionally, increasing severity of soft-tissue injury is associated with an appreciably higher rate of nonunion.^[19]^ The patellar tendon receives its blood supply from several sources, with the fat pad being responsible for the posterior portion and the retinacular and recurrent tibial arteries anteriorly, leaving the proximal and distal insertion areas relatively avascular and more susceptible to rupture.^[20,21]^ In our case report, given that the avulsion fracture of the tibial tubercle was relatively large, it was treated with a hollow screw associated with a tension band of steel wire. Notably, the site of tibial plateau nonunion was located the proximal end of the tibial tubercle. Therefore, we believe that the blood supply to the tibial plateau was disrupted because of fracture displacement and patellar tendon rupture, which in turn may have been a key factor resulting in the TPF nonunion in this case. Besides this, the patient presented with anemia and hypoproteinemia in our hospital, which often induces metabolic changes and influences fracture healing, and the articular cartilage and soft-tissue may have a lower healing capacity in older patients than in younger ones. Several studies conclude that segmental bone loss, open fractures, anemia, and hypoproteinemia are significant risk factors in the occurrence of nonunion^[22–24]^; however, a 2022 study found that the types of nonunions included septic (17.7%) and aseptic (82.3%) nonunions; in addition, nonvascular (86.5%) and vascular (13.5%) nonunions were seen within aseptic nonunions. Septic nonunion is associated with inflammation, younger age, and hypoproteinemia compared with anemia.^[18]^ Thus, we propose that severely comminuted or open proximal tibial fractures may be key factors in the nonunion of TPFs, especially for those combined with skin injury, patellar tendon disruption,anemia, and hypoproteinemia.

This case report has itslimitations. First, although, tibial plateau nonunion in this case was located at the distal end of the tibial tubercle, there is insufficient evidence to conclude that disruption of patellar tendon insertion blood supply played the important role in TPF nonunion. The possible reasons in our case include skin injury, anemia, and hypoproteinemia.^[25]^ As a result, the authors could not deduce what are the most important factors for the nonunion. Second, the surgical techniques of TPF, relevant articles in nonunions are relatively rare. There was insufficien data to help analyze other possible reasons and provide advice to explain the pathological mechanism of nonunions. Third, the lack of a universally accepted definition of a tibial plateau nonunion was evident; however, the definition of tibial plateau nonunion in this case report is in accordance with the criteria that established by the Food and Drug Administration, which defines a nonunion as “a fracture that is at least 9 months old and has not shown any signs of healing for 3 consecutive months.”^[11]^

## 4. Limitations

This study has several limitations. First, given that this was a retrospective case report, we did not photograph the internal fixator during the surgical procedure, which may negatively affect the reliability and authenticity of this report. All the clinical and radiographic data were collected from the same hospital. In addition, the patients were followed up for more than 2 years, and we have sufficient radiographic data to support the conclusion that nonunion may be a complication of an open complex tibial plateau fracture with patellar tendon rupture.

## 5. Conclusion

This report describes that severely comminuted or open proximal tibial fractures may be key factors in the nonunion of TPFs, especially for those combined with skin injury, patellar tendon disruption, anemia, and hypoproteinemia.

## Author contributions

**Conceptualization:** Wu Zeng.

**Data curation:** Wu Zeng.

**Funding acquisition:** Wu Zeng.

**Investigation:** Tao Xiong, Wu Zeng.

**Resources:** Tao Xiong.

**Supervision:** Zhou Feng Fan.

**Writing – original draft:** Tao Xiong.

**Writing – review & editing:** Zhou Feng Fan, Tao Xiong.
